# Preoperative gastric ultrasound in children with cerebral palsy: a cross-sectional observational study

**DOI:** 10.1016/j.bjane.2026.844728

**Published:** 2026-01-24

**Authors:** Cristiane de Pauli Bernardin, Juliana Thomaz Menck, Bruna Bastiani dos Santos, Jorge Eduardo Fouto Matias

**Affiliations:** a Hospital Pequeno Príncipe, Department of Anestesiology, Curitiba, PR, Brazil; b Universidade Federal do Paraná (UFPR), Curitiba, PR, Brazil; c Universidade Federal do Paraná(UFPR), Curitiba, PR, Brazil; d Universidade Federal do Paraná (UFPR), Department of Anestesiology, Curitiba, PR, Brazil

**Keywords:** Anesthesia, Cerebral palsy, Fasting, Preoperative period, Pyloric antrum, Ultrasound

## Abstract

**Background:**

Pulmonary aspiration during anesthesia, though rare, can be catastrophic. Gastric ultrasound provides an objective assessment of gastric contents and may be particularly relevant for children with Cerebral Palsy (CP), who are at risk of delayed gastric emptying.

**Methods:**

We conducted a cross-sectional study in a pediatric hospital including children scheduled for elective surgery per ASA fasting guidelines. Preoperative gastric ultrasound measured antral CSA in right lateral decubitus, and gastric volume was estimated using the Perlas formula. Fasting time, medication use, and clinical data were recorded. Group comparisons used Wilcoxon, Fisher’s exact, or Chi-Square tests; multiple linear regression adjusted for confounders.

**Results:**

Sixty-two children were studied: 30 with Cerebral Palsy (CP) and 32 controls. No patient exceeded the high-risk gastric volume threshold (1.5 mL.kg^-1^) and no surgeries were cancelled. CP patients had shorter fasting times (6.5 vs. 8.0 h; p < 0.001) and higher medication use (47% vs. 6.3%; p < 0.001). Gastric CSA (4.0 vs. 3.0 cm^2^; p < 0.001) and estimated gastric volume per kg (0.7 vs. 0.4 mL.kg^-1^; p < 0.001) were greater in CP. Multivariable models showed attenuation, but quantile regression confirmed higher lower CSA (+1.25 cm^2^; p = 0.007). Excluding medication users, CP remained associated with greater gastric volume.

**Conclusions:**

Children with cerebral palsy exhibit larger CSA and higher gastric volumes despite adequate fasting. Although clinically safe, these findings support the role of gastric ultrasound in preoperative risk assessment for this vulnerable group.

## Introduction

Pulmonary aspiration during anesthesia is a rare but catastrophic event, which can lead to a mortality rate of 9%.^1^ Preoperative fasting is essential to prevent complications of this nature. Over the years, fasting protocols have been updated and there has been a trend towards flexibility, especially in relation to ingested liquids.^2^, ^3^, ^4^, ^5^

Current guidelines encourage the ingestion of liquids without residues up to two hours before surgery, to reduce patient discomfort, catecholamine release and hemodynamic complications related to dehydration.^6^

The growth of portable ultrasound in surgical centers has sparked interest in its use as a diagnostic method to evaluate gastric contents. Measuring the Cross-Sectional Area (CSA) of the gastric antrum has proven useful for scenarios in which fasting is doubtful or there is some risk factor for pulmonary aspiration.^7^

The gastric residual volume that increases the risk of aspiration is considered to be above 1.5 mL.kg^-1^.^8^ Therefore, patients taking continuous medications could take them together with a glass of water up to two hours before surgery without compromising fasting. However, there is significant interindividual variation in gastric emptying, regardless of the duration of fasting, with some patients still presenting residual gastric content even after prolonged fasting.^9^

Despite the current trend towards reducing fasting time for clear liquids recommended by several international pediatric societies;^2^ other societies continue to recommend traditional fasting times.^6^ This conflict in the literature has raised questions and many studies are underway to try to clarify the ideal fasting time for different populations.

Patients with cerebral palsy represent a group of individuals who may benefit from advances in this specific research area. They are considered a challenge in anesthetic induction, as they may have delayed gastric emptying and are frequently consuming anticonvulsants and muscle relaxants that should not be discontinued.^10^

This study was designed to estimate gastric content and volume by ultrasonography in children with and without cerebral palsy undergoing elective surgical procedures and who followed the traditional preoperative fasting strategy recommended by the American Society of Anesthesiology (ASA).^6^

Also, we wanted to verify whether patients with cerebral palsy require a preoperative fasting period longer than recommended, in order to increase safety of anesthetic induction in this group of individuals.

## Methods

The study was performed in an exclusive pediatric hospital, and was submitted to and approved by the research Ethics Committee and registered under number CAAE 52573121.0.0000.0097. Patients were selected on the day of surgery in the admission room of the surgical theater and after the application of the informed consent form. Data collection was performed at a single time before anesthetic induction by the same trained operator, according to the standard scanning protocol,^11^ no premedication was used. The period of recruitment was 12 months. This article followed EQUATOR Reporting Guidelines.

Children under 18 years of age, with or without cerebral palsy scheduled for elective surgical procedures were selected. All patients without cerebral palsy were ASA I class (without comorbidities or healthy). We excluded patients with previous gastrointestinal surgery, gastric tubes or stomas, anatomical abnormalities of the gastrointestinal tract and pregnancy.

All patients followed the preoperative fasting guideline recommended by the ASA.^3^

A convenience sample of 30 patients with cerebral palsy and 32 healthy patients was recruited representing the two groups of study.

The use of medications was not an exclusion criterion due to the difficulty of sampling without their use. No formal a priori sample size calculation was performed. The chosen sample size was based on feasibility within the study period.

### Ultrasound assessment

All ultrasound scans were performed in the morning to avoid prolonged fasting, with the same ultrasound device (M-turbo Sonosite) and a low-frequency curved transducer (2 to 6 MHz) by a single operator. Patients were first scanned in the Supine Position (SP) and then in the Right Lateral Decubitus (RLD). The transducer was placed in the sagittal plane at the epigastric region to visualize the gastric antrum between the left hepatic lobe and the pancreas at the level of the aorta. The cross-sectional area of the gastric antrum (CSA) was calculated using the RLD ultrasound image, based on the two-diameter Craniocaudal (CC) and Anteroposterior (AP) method formula as previously described by Perlas et al.:^8^ CSA=(APxCCxπ)/4.

Qualitative assessment of the gastric content was initially performed, resulting in three possibilities: 1) Empty-anterior and posterior walls juxtaposed; 2) Hypoechoic liquid content or 3) Solid content, distended lumen with a “ground glass” appearance. Then, the total Volume (V) of gastric fluid was estimated in milliliters using the mathematical model validated and suggested by Perlas et al., through the following formula:^8^ V=27.0+14.6×CSAinRLD−1.28×age; where: Volume in milliliters (mL); CSA in right lateral decubitus in cm^2^; Age in full number of years.

Parameter data were recorded on an electronic spreadsheet where individual data regarding age, sex, weight, type of surgery, fasting time and continuous medications were also recorded.

For each patient, fasting intervals for solids and clear liquids were recorded separately, in accordance with ASA guidelines. These variables were analyzed both as continuous values (hours) and categorical thresholds (< 8 h vs. ≥ 8 h), for solids and liquids independently.

### Statistical analysis

Continuous variables were summarized as medians with Interquartile Ranges (IQR) and compared between groups using the Wilcoxon rank-sum test. Categorical variables were compared using Fisher’s exact test or the Chi-Squared test, as appropriate. The primary outcome was the gastric antral Cross-Sectional Area (CSA). Between-group differences in CSA were estimated using the Hodges-Lehmann method for the median difference, with 95% Confidence Intervals (95% CI). To adjust for potential confounding, a multivariable linear regression model was fitted including age, weight, fasting time (in hours, as a continuous variable), and continuous medication use. Model assumptions were verified through residual diagnostics, and robust (HC3) standard errors were applied when heteroscedasticity or non-normality was identified. Sensitivity analyses were performed using quantile regression to explore the effect of group status across the distribution of CSA, rather than focusing solely on the mean. In addition, estimated gastric volume (mL) and weight-adjusted volume (mL.kg^-1^), derived from established ultrasound-based equations, were evaluated as secondary complementary outcomes to provide clinically interpretable measures. All tests were two-sided, and a p-value < 0.05 was considered statistically significant.

## Results

The study included 62 patients, 30 with cerebral palsy and 32 without cerebral palsy. No patient presented solid or liquid content above 1.5 mL.kg^-1^; therefore no one had an “at-risk stomach”. No surgical procedures were postponed or canceled. The most common interventions were application of botulinum toxin in the cerebral palsy patients and circumcision in the healthy ones.

Half of the patients in the cerebral palsy group were users of anticonvulsant medications and muscle relaxants such as Baclofen, carbamazepine, phenobarbital, cannabidiol, valproic acid, quetiapine, risperidone, lamotrigine (summarized in Supplementary Table).

Baseline characteristics are summarized in Table 1. Median age and weight were comparable between groups. However, fasting duration was significantly shorter in children with cerebral palsy (6.5 vs. 8.0 h, p < 0.001), and continuous medication use was more frequent (47% vs. 6.3%, p < 0.001). The gastric antral Cross-Sectional Area (CSA) was significantly larger in the cerebral palsy group (median 4.0 vs. 3.0 cm^2^, p < 0.001), as was the estimated gastric volume per kilogram (0.7 vs. 0.4 mL.kg^-1^, p < 0.001). Visible residual fluid in the right lateral decubitus position was observed only in the cerebral palsy group (13%, p = 0.049).

**Table 1:** Clinical and demographic characteristics of the study population.

Variable	Healthy controls (n = 32)	Cerebral palsy (n = 30)	p-value
Age, years, median (IQR)	6.5 (3.0–12.0)	9.0 (6.0–12.0)	0.10
Weight, kg, median (IQR)	31.0 (18.0–42.5)	29.0 (18.0–40.0)	0.90
Fasting time, h, median (IQR)	8.0 (8.0–9.5)	6.5 (2.0–8.0)	< 0.001
Gastric CSA, cm^2^, median (IQR)	3.0 (2.0–4.0)	4.0 (4.0–5.0)	< 0.001
Volume, mL.kg^-1^, median (IQR)	0.4 (0.3–0.6)	0.7 (0.6–0.9)	< 0.001
Visible fluid (right lateral)	0 (0%)	4 (13%)	0.049
Continuous medication use, n (%)	2 (6.3%)	14 (47%)	< 0.001

CSA, Cross-Sectional Area; IQR, Interquartile Range.

In the multivariable linear regression model for CSA (Table 2), cerebral palsy was associated with a mean CSA increase of +0.92 cm^2^ after adjustment for age, weight, fasting time, and medication use. This effect did not reach statistical significance (95% CI -0.27 to 2.10, p = 0.13). None of the covariates showed significant independent associations with CSA.

**Table 2:** Multivariable linear regression for gastric CSA.

Characteristic	Beta (cm^2^)	95% CI	p*-*value
Group (cerebral palsy vs. control)	+0.92	−0.27 to 2.10	0.13
Age (years)	−0.02	−0.24 to 0.19	0.80
Weight (kg)	+0.02	−0.05 to 0.10	0.60
Fasting time (h)	−0.07	−0.28 to 0.13	0.50
Continuous medication (yes vs. no)	+0.96	−0.54 to 2.50	0.20

CSA, Cross-Sectional Area; CI, Confidence Interval.

Quantile regression results are presented in Table 3. The effect of cerebral palsy on CSA was most pronounced at the lower quartile (τ = 0.24), with an estimated difference of +1.25 cm^2^ (95% CI 0.36 to 2.16, p = 0.007). At the median (τ = 0.50) and upper quartile (τ = 0.75), the differences were smaller and not statistically significant. These findings suggest that children with cerebral palsy consistently avoided the very low CSA values observed in controls.

**Table 3:** Quantile regression for gastric CSA.

Quantile (τ)	Estimate (cm²)	95% CI	p-value
0.25	+1.26	0.36 to 2.16	0.007
0.50	+0.68	−0.28 to 1.64	0.16
0.75	+1.00	−0.66 to 2.66	0.23

CSA, Cross-Sectional Area; CI, Confidence Interval.

For estimated gastric volume (Table 4), the unadjusted comparison showed significantly higher values in the cerebral palsy group (median 23 vs. 10.5 mL, p < 0.001). However, in the adjusted model, the group effect was attenuated (+1.9 mL, 95% CI -4.0 to 7.8, p = 0.50). Age was strongly associated with increased gastric volume (+2.9 mL.year^-1^, 95% CI 1.8 to 4.0, p < 0.001), whereas fasting time and medication use did not reach significance.

**Table 4:** Multivariable linear regression for estimated gastric volume.

Characteristic	Beta (mL)	95% CI	p-value
Group (cerebral palsy vs. control)	+1.9	−4.0 to 7.8	0.50
Age (years)	+2.9	1.8 to 4.0	< 0.001
Weight (kg)	−0.25	−0.63 to 0.12	0.20
Fasting time (h)	−0.88	−1.9 to 0.13	0.087
Continuous medication (yes vs. no)	+3.5	−4.0 to 11	0.40

CI, Confidence Interval.

A subgroup analysis excluding children on continuous medication (16 cerebral palsy vs. 30 controls) showed that, with comparable fasting times and no gastroparesis-inducing drugs, cerebral palsy was associated with an 8–10 mL higher gastric volume (p = 0.03) (Supplementary Table).

Descriptive statistics and group comparisons are shown in Table 4. Median fasting time and type of intake differed significantly across groups (p < 0.001). Children with CP using medications presented markedly shorter fasting times and larger gastric antral Cross-Sectional Areas (CSA) and estimated gastric volumes per kilogram. Significant group differences were confirmed for both CSA and gastric volume.kg^-1^ (p < 0.001). Visible fluid in the right lateral decubitus position was observed only in the medicated CP group (p = 0.002).

Table 5 Descriptive characteristics of the study population by group. An additional comparative analysis including three distinct groups: 1) Healthy controls, 2) Children with Cerebral Palsy (CP) using medications known to affect gastric emptying, and 3) Children with CP not using such medications.

**Table 5:** Descriptive characteristics of the study population (three-group comparison).

Variable	Healthy controls (n = 32)	Cerebral palsy – With medication (n = 14)	Cerebral palsy – Without medication (n = 16)	p-value^a^
**Age (years)**	6.5 (3.0 – 12.0)	9.0 (6.0 – 11.0)	10.0 (5.5 – 12.0)	0.20
**Weight (kg)**	31.0 (18.0 – 42.5)	28.0 (20.0 – 38.0)	31.5 (18.0 – 42.0)	0.90
**Fasting time (hours)**	8.0 (8.0 – 9.5)	2.0 (2.0 – 2.0)	8.0 (7.5 – 8.0)	< 0.001
**Type of last intake**				< 0.001
Breast milk or formula	0 (0 %)	1 (7.1 %)	3 (19 %)	
Clear liquid	0 (0 %)	11 (79 %)	1 (6.3 %)	
Solid food	32 (100 %)	2 (14 %)	12 (75 %)	
**Gastric CSA (cm^2^)**	3.0 (2.0 – 4.0)	4.5 (4.0 – 7.0)	4.0 (3.5 – 4.5)	< 0.001
**Estimated gastric volume (mL.kg^-1^)**	0.4 (0.3 – 0.6)	0.7 (0.6 – 1.4)	0.6 (0.5 – 0.8)	< 0.001
**No visible fluid (supine)**	32 (100 %)	14 (100 %)	16 (100 %)	–
**No visible fluid (right lateral)**	32 (100 %)	10 (71 %)	16 (100 %)	0.002

a Kruskal-Wallis rank-sum test for continuous variables; Fisher’s exact test for categorical variables. Data are median (Q1, Q3) or number (%). CSA, Cross-Sectional Area.

## Discussion

It is well known that prolonged fasting (over 24 hours) can delay gastric emptying.^12^ According to the most current protocols, early refeeding in the postoperative period, as well as a minimum period of preoperative fasting, is ideal.^2^^,^^4^^,^^13^

In addition to fasting, the use of ultrasound has improved the safety and quality of anesthesia by guiding the adoption of more appropriate strategies to reduce aspiration and by assessing the nature and volume of gastric contents.^7^^,^^14^^,^^15^ With adequate training, the success rate in the evaluation and correct measurement of the gastric antrum can reach 95%.^15^^,^^16^

Cerebral palsy is a permanent condition of delayed neuropsychomotor development attributed to non-progressive fetal or early childhood disorders.^17^^,^^18^ Furthermore, delayed gastric emptying in patients with cerebral palsy has been reported even with adequate fasting.^19^

In this series, cerebral palsy patients presented a higher antrum CSA than healthy patients and consequently a larger estimated gastric volume.

However, when we inferred about gastric emptying by correcting the variables for fasting time, age and medication use, this difference was not statistically significant, probably due to the limited sample size. Adjusted analysis indicates that part of the higher CSA observed in children with cerebral palsy is associated with confounding factors (especially fasting time and medication use), as controlling for these factors reduced the significance of the difference between the groups. However, robust statistical methods indicate that the difference in CSA still persists, suggesting that the neurological condition itself possibly contributes to greater gastric filling independently of these factors.

Although children with cerebral palsy presented more fluid in the lateral decubitus position, this was associated with the use of continuous medications and shorter fasting time. Children with cerebral palsy and their parents were advised not to discontinue anticonvulsants and muscle relaxants before procedures resulting in shorter fasting time, which was a confounding factor in half of the children of this group.

No child had a full stomach (solid or liquid content above 1.5 mL.kg^-1^). This may be attributable to the rigorous preoperative fasting and it aligns with results of similar studies in the recent literature.^20^

One limitation of dealing with this disease is that there are several degrees of impairment in cerebral palsy; some patients are completely dependent on mechanical ventilation, and other patients are able to walk and feed themselves without the help of tubes or stomas.^21^ This variability in the degrees of impairment of cerebral palsy may have led to no difference being initially found in the groups, since the most severe cases were excluded for being users of gastrostomy.

In a recent study, it was not possible to correlate the severity of the disease and the increase in gastric volume,^20^ possibly due to the same limitation of number and homogeneity present in the sample of our study.

Furthermore, the mathematical formula used to estimate gastric volume has not yet been validated in children, especially in those with cerebral palsy, who may have distortions in their results since, compared to healthy children of the same age, they are smaller.^21^

There was difficulty in increasing the sample of patients with cerebral palsy because it is an uncommon condition, and the most severe patients with gastrostomies and gastric tubes were excluded from the study. In addition, during the data collection period, although some patients had returned to the hospital to undergo a new procedure, they were not included again in the sample.

The use of gastric ultrasound in this specific area of clinical investigation still requires some standardization, including, for example, the definition of minimum training requirements for anesthesiologists to ensure accurate assessments.^15^ The gold standard for more accurate assessment continues to be scintigraphy,^22^ however, its use in a preoperative scenario is unfeasible. In addition, most of the data currently published refer to healthy adults. The volume assessment models, in particular, have only been validated for adult and non-pregnant populations, and more data are needed from pediatric populations.^23^

A subgroup analysis excluding children on continuous medication suggests that medication use and shorter fasting partially masked the effect of the neurological condition. When controlled, cerebral palsy itself impacted residual gastric volume, likely due to reduced gastrointestinal motility. Despite reduced sample size and potential selection bias, the evidence supports a genuine effect of cerebral palsy on increasing gastric volume.

The three-group comparative analysis confirmed that the use of medications known to delay gastric emptying is associated with shorter fasting times and higher gastric volume. However, even among non-medicated children with cerebral palsy, gastric volume remained higher than in healthy controls, suggesting that the neurological condition itself contributes to altered gastric motility independently of pharmacologic effects.

Possibly a larger and more homogeneous sample, excluding the use of medications that can affect gastric emptying, could show a more reliable result regarding the neurological disease and its impact on the digestive tract.

The major difficulty in carrying out this type of work was obtaining a relatively large, homogeneous sample, free of confounding factors such as the use of medications, surgeries or malformations of the gastrointestinal tract.

Our results reinforce the use of tools such as ultrasound in special cases, such as pregnant women, diabetics, and those with neurological and digestive tract diseases, since there is no harm to the patient in applying this method.^7^

3D and 4D ultrasound are newer imaging modalities that may play a future role in gastric ultrasound evaluation and bring greater safety to clinical practice.^24^

## Conclusion

Pediatric patients with cerebral palsy demonstrated significantly larger gastric antral CSA and higher estimated gastric volumes than healthy controls, despite adherence to preoperative fasting guidelines. Although none exceeded the threshold for aspiration risk, these findings suggest that neurological impairment itself may contribute to increased residual gastric content, and these patients may require individualized preoperative fasting strategies, incorporating adjunctive tools such as gastric ultrasound. Larger studies, including patients across the full spectrum of cerebral palsy severity, are warranted to confirm these results and refine clinical recommendations.

## Data availability statement

The datasets generated and/or analyzed during the current study are available from the corresponding author upon reasonable request.

## AI assistance disclosure

The authors declare that no Artificial Intelligence (AI) tools were used in the preparation, writing, language editing, or analysis of this manuscript. The authors take full responsibility for the integrity and accuracy of its content.

## Funding

This study was supported by a scholarship from the Surgical Department of the Federal University of Paraná (CAPES–DS agency, government program, grant number 40001016018P0).

## Conflicts of interest

The authors declare no conflicts of interest.

## Appendix Supplementary materials

Image, application 1

## Footnotes

Supplementary material associated with this article can be found in the online version at doi:10.1016/j.bjane.2026.844728.
