# Supplementary material for: Preoperative gastric ultrasound in children with cerebral palsy: a cross-sectional observational study
Source: Braz J Anesthesiol. 2026 Jan 24;76(3):844728. doi: 10.1016/j.bjane.2026.844728 (PMC12993129; doi:10.1016/j.bjane.2026.844728)
Supplement: Supplementary file 1 [file mmc1.docx]

**BJAN-D-25-00332_Supplementary Material**

**Supplementary Table 1** Comparison of patients without medication use, with and without cerebral palsy.

| **Characteristics** | **Healthy Controls**  **(n = 30)** | **Cerebral Palsy**  **(n = 16)** | **p-value^b^** |
| --- | --- | --- | --- |
| Weight, kg^a^ | 31.0 (17.5–41.5) | 31.5 (18.0–41.0) | 0.817 |
| Fasting time |  |  | 0.011 |
| ≥ 8 h | 30 (100.0) | 12 (75.0) |  |
| < 8 h | 0 (0.0) | 4 (25.0) |  |
| Supine position |  |  |  |
| No fluid | 30 (100.0) | 16 (100.0) |  |
| Lateral position |  |  | > 0.999 |
| No fluid | 30 (100.0) | 16 (100.0) |  |
| Fluid present | 0 (0.0) | 0 (0.0) |  |
| Age, years^a^ | 6.5 (3.3–12.0) | 10.0 (5.8–12.0) | 0.198 |
| ACT^a^ | 3.0 (2.0–4.0) | 4.0 (3.8–4.3) | 0.027 |
| Estimated volume, mL^a^ | 10.5 (7.0–19.0) | 21.5 (13.5–26.0) | 0.018 |
| Medication use | 0 (0.0) | 0 (0.0) |  |

^a^ Median (IQR); n (%).

^b^ Wilcoxon rank-sum test; Fisher’s exact test.

**Supplementary Table 2** Linear regression for estimated gastric volume in patients without medication use.

| **Characteristics** | **Beta (95% CI)** | **p-value** |
| --- | --- | --- |
| Fasting time < 8 h | 3.4 (-6.8 to 13.6) | 0.502 |
| Cerebral palsy | 6.5 (0.5 to 12.5) | 0.035 |

CI, Confidence Interval.

**Supplementary Table 3** Frequency of medications observed in the cerebral palsy group (n = 20).

| **Medication** | **n (%)** |
| --- | --- |
| Baclofen | 9 (45.0) |
| Carbamazepine | 3 (15.0) |
| Valproate | 2 (10.0) |
| Atropine (sublingual) | 1 (5.0) |
| Cannabidiol | 1 (5.0) |
| Phenobarbital | 1 (5.0) |
| Lamotrigine | 1 (5.0) |
| Quetiapine | 1 (5.0) |
| Risperidone | 1 (5.0) |

¹n (%).
